# Supplementary material for: Complexome profiling of the Chlamydomonas psb28 mutant reveals TEF5 as an early PSII assembly factor
Source: Plant Cell. 2025 Mar 17;37(6):koaf055. doi: 10.1093/plcell/koaf055 (PMC12164590; doi:10.1093/plcell/koaf055)
Supplement: koaf055_Supplementary_Data [file koaf055_supplementary_data.zip › psb28_interactive.zip!/plots/intensity/qualityPlots/peptides/psbC_Cre-1.g2716963.t1.p1.html]

### Cre-1.g2716963.t1.p1

Trivial name: psbC  
SeqCoverage [median]: 40.80  
Mass: 50.64 kDa  
  
 

| Sequence | MissedCleavage | Mass [Da] | wtIntensity | muIntensity | Score |
| --- | --- | --- | --- | --- | --- |
| AAAAGFEK | 0 | 763.39 | 1582870.19 | 325711.70 | 157.21 |
| AAAAGFEKGIDRFDEPVLSMRPLD | 3 | 2604.30 | 0.00 | 31081.09 | 78.92 |
| AAEYMTHAPLGSLNSVGGVATEINAVNFVSPR | 0 | 3271.63 | 245509.56 | 0.00 | 135.02 |
| AMYFGGVYDTWAPGGGDVR | 0 | 2017.90 | 70767.31 | 1728.57 | 188.09 |
| ARAAAAGFEK | 1 | 990.52 | 1171.43 | 0.00 | 88.50 |
| DQETTGFAWWSGNAR | 0 | 1724.75 | 179874.34 | 0.00 | 299.20 |
| DQRLGANVASAQGPTGLGK | 1 | 1838.96 | 672.86 | 2442.98 | 48.38 |
| FDEPVLSMRPLD | 1 | 1417.69 | 83520.16 | 16462.64 | 178.54 |
| FWDFRGPWLEPLR | 1 | 1717.87 | 2477.83 | 0.00 | 71.61 |
| GIDRFDEPVLSMR | 1 | 1533.76 | 16895.05 | 3641.62 | 96.87 |
| GIDRFDEPVLSMRPLD | 2 | 1858.92 | 2983213.12 | 1180283.41 | 199.33 |
| GPNGLDLNK | 0 | 926.48 | 239701.85 | 76589.46 | 239.66 |
| GPNGLDLNKLK | 1 | 1167.66 | 0.00 | 744.13 | 33.88 |
| GPWLEPLR | 0 | 966.53 | 89820.46 | 0.00 | 127.12 |
| GPWLEPLRGPNGLDLNK | 1 | 1875.00 | 100700.98 | 9747.88 | 130.21 |
| LGANVASAQGPTGLGK | 0 | 1439.77 | 3768321.75 | 545496.51 | 435.11 |
| LGANVASAQGPTGLGKYLMR | 1 | 2003.06 | 0.00 | 10474.44 | 73.21 |
| LINLSGK | 0 | 743.45 | 2175063.69 | 305880.13 | 172.03 |
| LKNDIQPWQER | 1 | 1425.74 | 789559.24 | 131459.94 | 165.09 |
| LKNDIQPWQERR | 2 | 1581.84 | 56834.11 | 34307.79 | 128.28 |
| NDIQPWQER | 0 | 1184.56 | 218302.08 | 36435.16 | 244.81 |
| NDIQPWQERR | 1 | 1340.66 | 30958.55 | 23908.22 | 88.50 |
| RAAEYMTHAPLGSLNSVGGVATEINAVNFVSPR | 1 | 3427.73 | 89842.79 | 0.00 | 171.31 |
| SPTGEIIFGGETMR | 0 | 1493.72 | 1506158.50 | 481154.92 | 307.55 |
| SPTGEIIFGGETMRFWDFR | 1 | 2245.06 | 10621.29 | 0.00 | 93.77 |
| VITNPTTNAAVIFGYLVK | 0 | 1920.07 | 1376257.13 | 27993.98 | 305.60 |
| YLMRSPTGEIIFGGETMR | 1 | 2057.01 | 0.00 | 1435.47 | 47.75 |
|  |  |  | 15619114.3 | 3246980.1 |  |
